# Spin pumping in Ferromagnet-Topological Insulator-Ferromagnet Heterostructures

**DOI:** 10.1038/srep07907

**Published:** 2015-01-20

**Authors:** A. A. Baker, A. I. Figueroa, L. J. Collins-McIntyre, G. van der Laan, T. Hesjedal

**Affiliations:** 1 Department of Physics, Clarendon Laboratory, University of Oxford, Oxford, OX1 3PU, United Kingdom; 2 Magnetic Spectroscopy Group, Diamond Light Source, Didcot, OX11 0DE, United Kingdom

## Abstract

Topological insulators (TIs) are enticing prospects for the future of spintronics due to their large spin-orbit coupling and dissipationless, counter-propagating conduction channels in the surface state. However, a means to interact with and exploit the topological surface state remains elusive. Here, we report a study of spin pumping at the TI-ferromagnet interface, investigating spin transfer dynamics in a spin-valve like structure using element specific time-resolved x-ray magnetic circular dichroism, and ferromagnetic resonance. Gilbert damping increases approximately linearly with increasing TI thickness, indicating efficient behaviour as a spin sink. However, layer-resolved measurements suggest that a dynamic coupling is limited. These results shed new light on the spin dynamics of this novel material class, and suggest great potential for TIs in spintronic devices, through their novel magnetodynamics that persist even up to room temperature.

The exciting physics of topological insulators (TIs) has been under intense study since their theoretical prediction^1^ and experimental verification^2^,^3^,^4^. Recently, they were shown to display the quantum anomalous Hall effect after doping with magnetic impurities^5^, and are proposed to host image magnetic monopoles and the giant magneto-optical effect^6^,^7^,^8^,^9^. In the prototypical three-dimensional TI Bi_2_Se_3_ a large spin-orbit interaction leads to a band inversion in the bulk and the formation of a topologically protected surface state (TSS), with fully spin-polarised counter-propagating conduction channels that are robust against scattering from non-magnetic impurities^10^. Figure 1a shows a diagram of the bandstructure of a TI. Spin-momentum locking suggests the possibility of very long spin-flip scattering lifetimes and the ability to generate ultra-high spin-orbit torques^11^,^12^,^13^. It has been predicted that the TSS can exert a torque on spins in a neighbouring ferromagnet (FM) through exchange coupling^14^. However, in order to realise these prospects the magnetodynamics of the TSS must be studied and such TI-FM heterostructures fabricated. While angle-resolved photo-emission spectroscopy has been extremely successful at identifying TIs^3^,^4^, transport measurements have met with limited success due to large bulk conductivities that make unambiguously identifying the surface state challenging^15^,^16^. It is therefore highly desirable to apply a wider range of techniques to the study of TIs, aiming to focus more closely on the spin degrees of freedom present in the TSS.

A key focus of spintronic research in recent years has been the phenomenon of spin pumping^17^,^18^, whereby the ferromagnetic resonance (FMR) generates a pure spin current that enters adjacent layers^19^. Such non-local spin dynamics manifest as an additional damping term in the Landau-Lifshitz-Gilbert (LLG) equation for magnetodynamics^20^, broadening the measured resonance. Furthermore, spin pumping through a nonmagnetic spacer into a second FM affects the phase and amplitude of precession^21^, allowing direct confirmation of the presence of a coherent pure spin current. The application of this technique to TIs is quite naturally suggested by the similarity between the spin-locked surface state of a TI and the separation of angular momentum and charge flow that takes place in a pure spin current. Recently, studies of such exciting effects have begun to emerge through electrical transport and inverse spin-hall effect measurements^13^,^22^,^23^, demonstrating the great potential of TIs for incorporation into spintronic devices such as spin valves.

In this letter we use FMR and time-resolved x-ray magnetic circular dichroism (TR-XMCD) to study the spin pumping in FM-TI-FM heterostructures. TR-XMCD allows element- (and thus layer-) specific detection of precession of magnetisation^21^,^24^,^25^. The phase of precession across magnetic resonances is highly sensitive to spin transfer phenomena. The ability of a TI to absorb and transfer a pure spin current is probed, determining the spin mixing conductance. We find that the TI interlayer functions as an excellent spin sink, dramatically increasing the Gilbert damping of magnetodynamics in the FM layers. However, there is only weak evidence of spin transfer through the TI to a second FM, which could be explained either by dynamic exchange or by a short range coupling between surface states that is suppressed with increasing TI thickness.

In the classical limit the dynamics of magnetization can be described by the LLG equation of motion, which represents the precessional torque arising due to an effective internal field, ***H***_eff_, and the damping due to the phenomenological Gilbert torque, *α*. Gilbert damping includes energy loss mechanisms such as spin-flip scattering and the excitation of phonons. This equation can be readily modified to include spin pumping; for a trilayer structure with only one layer, *i*, on resonance, the motion of magnetisation ***m****_i_*, is^18^:
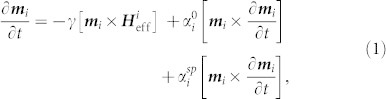
where the subscript denotes the magnetic layer number, *γ* is the gyromagnetic ratio, *α*^0^ the intrinsic Gilbert damping parameter and *α*^sp^ additional damping due to spin pumping. The third term represents increased damping in layer *i* due to spin pumping. If the second layer is allowed to precess an anti-damping (or accelerating) torque from the STT induced by momentum transfer from layer *j* is also present, but this vanishes if the resonances are well separated in frequency-field space. This assumption is valid in all samples considered here, as confirmed by the TR-XMCD measurements. The additional damping associated with spin pumping can be written as^21^:
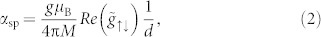
where *g* is the Landé *g*-factor, *μ*_B_ the Bohr magneton, *M* the magnetisation of the magnetic layer, *d* the layer thickness and 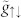 the spin mixing conductance, which controls spin-selective transport across the interface^17^. Spin pumping across a non-magnetic barrier usually decays exponentially, as scattering of the pure spin current leads to a loss of angular momentum and backflow into the on-resonance layer. This is in effect a simultaneous damping and anti-damping of motion, for thinner barriers the second ferromagnet absorbs the spin current and minimises backflow, while for thicker barriers spin pumping is reduced as more electrons are scattered. See the Supplementary Information for more theory of spin pumping in normal metals and TIs.

A schematic of the heterostructure is shown in Fig. 1b. Samples were prepared by molecular beam epitaxy, on MgO(001) substrates. Growth was monitored using in-situ reflection high energy electron diffraction (RHEED), see Fig. 1c. First, 30 nm of Co_50_Fe_50_ were deposited and annealed to form an epitaxial ferromagnetic layer. Next, Bi_2_Se_3_ was deposited, with thickness ranging from 4 nm to 20 nm. 30 nm of Ni_81_Fe_19_ were then deposited at room temperature (300 K) forming a polycrystalline layer, as demonstrated by rings in the RHEED pattern. This is important in order to prevent damage to the TI due to Se out-diffusion or Fe intercalation. See Supplementary Information for more details.

Vector network analyser (VNA) FMR measurements were performed in the frequency range 0.5–20 GHz for samples mounted on a coplanar waveguide (CPW). The forces acting on a ferromagnet undergoing FMR are shown in Fig. 2a. Real and imaginary components of the microwave transmission parameter, *S*_12_, were measured as a function of bias field for multiple frequencies. Figure 2b shows a typical field-frequency-transmission map. The linewidth of the resonance is frequency (*f*) dependent and relates to the damping as^26^:
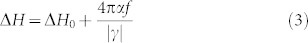
where Δ*H* is the full width at half maximum of the resonance, Δ*H*_0_ the inhomogeneous broadening arising from non-Gilbert damping mechanisms such as lattice defects and *α* = *α*^0^ + *α*^sp^ the Gilbert damping, including contributions related to coupling to the lattice (such as spin flip scattering, phonon drag or spin pumping).

Figure 2c shows the measured damping parameter, *α*, for the Ni_81_Fe_19_ and Co_50_Fe_50_ layers as a function of the thickness of the TI interlayer. A linear increase is observed up to 20 nm, wherein the spin pumping component represents a significant fraction of the total damping, indicating a large transfer of angular momentum to the TI. It is important to compare this result with other materials. Damping in trilayers normally drops exponentially with spacer thickness^27^, as scattering within the non-magnetic (NM) layer is a much less efficient sink for angular momentum than absorption by a second ferromagnet. Here, however, damping increases with TI thickness, suggesting not only that the TI is a very efficient spin-sink, but that the pure spin current can penetrate some distance into the TI, at least 8 nm (compared to ~ 3 nm in Si or <1 nm in Ta^21^,^28^).

As a spin current is driven into the TSS a spin imbalance develops, which is converted to a charge current^13^. The scattering of such conduction electrons within the bulk of the TI could then provide a mechanism for efficient absorption of pumped angular momentum.

It is instructive to analyse the spin pumping results in the analytical framework of the STT in normal metals. Equation 2 can be rearranged to yield the spin mixing conductance, 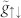. Considering, for example, the case of the Ni_81_Fe_19_ (M*_s_* = 0.906 × 10^6^ A/m) layer in the *t*_TI_ = 20 nm sample, the spin pumping damping can be extracted by comparison with a bare Ni_81_Fe_19_ layer, yielding *α*_sp_ = (2.6 ± 0.3) × 10^−3^. The spin mixing conductance is then 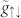 = (4.2 ± 0.5) × 10^15^ cm^−2^. Performing the same calculation for the Co_50_Fe_50_ layer in this sample (M*_s_* = 1.76 × 10^6^ A/m) gives 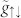 = (2.49 ± 0.1) × 10^15^ cm^−2^. The discrepancy between the results for the two layers points towards dissimilar interfaces. This most likely arises due to the requirement for low temperature deposition of the Ni_81_Fe_19_ layer, in order to preserve crystal quality within the Bi_2_Se_3_ layer.

Since the damping does not completely saturate these value should be regarded only as a lower limit on the effective spin mixing conductance available in FM/TI heterostructures. These values are comparable to the spin mixing conductances calculated by Jamali *et al.* from their inverse spin Hall effect measurements^29^. They are greater than previous reports for even a good spin conductor such as Ag, where 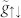 ≈ 2 × 10^15^ cm^−2^ 30. Note that the calculation does not separate pumping into the bulk and surface state, which display very different spin dynamics. The value should therefore be considered only as a rough estimate of the effective spin mixing conductance that is available in TI heterostructures.

TR-XMCD allows element specific measurement of the precession of magnetisation within each ferromagnetic layer through polarisation dependence of x-ray absorption at the *L*_2,3_ edges^31^. A resolution of several picoseconds is achieved through synchronisation of x-ray bunch arrival with the RF driving precession. The layer-specific phase of precession is determined with high precision by analysing the amplitude of the XMCD signal, making it a powerful tool to study the subtle effects of coupling through the TI. Figure 3a shows a schematic of the measurement geometry.

Precession of Ni magnetisation is shown in Fig. 3b for the Co_50_Fe_50_(30)/Bi_2_Se_3_(8)/Ni_81_Fe_19_(30) sample (thicknesses in nm). The increase in amplitude of precession and phase shift is clearly visible across the resonant field of 14 mT. Fitting sine curves to the precession yields amplitude and phase, which allows study of the coupling of the two layers.

Figure 4 shows the phase of precession determined in this way for three different thicknesses of interlayer. In a traditional spin valve, coupling of the two magnetic layers is mediated by the passage of a pure spin current through the spacer, leading to off resonance precession and a phase shift. Such features can be seen in the Ni_81_Fe_19_ layer for *t*_TI_ = 8 nm (Fig. 4b) and the Co_50_Fe_50_ layer for *t*_TI_ = 4 nm (Fig. 4a). However, this effect is rather weak, suggesting a suppression of spin pumping due to spin-flip scattering. Measurements on a sample with 20 nm Bi_2_Se_3_ (not shown) showed no evidence of coupling, suggesting complete absorption of the pumped spin angular momentum. This confirms that the weak dynamic coupling of the two layers is suppressed at this thickness, suggesting that the pumped spin current is scattered by the maximum thickness measured. We note that good conductors such as Cu, Ag or Au can have a spin coherence length of tens of nanometres^27^, while trivial insulators such as MgO or heavy metals such as Ta suppress spin pumping after just a few nm^21^,^32^. Suppression of spin pumping in insulators was recently studied by Du *et al.*^28^, who showed that there was a characteristic decay length of under 1 nm for SrTiO_3_, Sr_2_GaTaO_6_ and Sr_2_CrNbO_6_. Amorphous silicon also suppressed spin pumping for films 3 nm thick. As a heavy element bulk bandgap insulator Bi_2_Se_3_ might therefore be expected to suppress spin pumping, absorbing angular momentum.

The TR-XMCD results must therefore be considered alongside the VNA-FMR measurements. First, it is important to consider the distinction between the two techniques: VNA-FMR measures increased damping due to spin pumping out of a FM, while TR-XMCD allows detection of modified precessional dynamics induced by spin pumping into a FM. The increase of damping parameter with TI thickness suggests that the TI functions as an excellent spin sink, but transfer of the spin current is less efficient. The weak coupling observed in the TR-XMCD could be attributed to transmission of a pure spin current, with a decay length on the order of 8 nm. While the current can persist within the TI (as demonstrated by continued increase of Gilbert damping with TI thickness), its passage across the FM/TI interface and the topological surface state is suppressed. The dissimilar interfaces indicated by the calculated values of 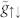 suggest that this transmission could be improved (and even higher values of 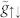 obtained) if the quality of the interface in future devices can be improved.

An alternative explanation could arise from the proximity coupling of 'top' and 'bottom' surface states that takes place in ultrathin TIs. As shown by Zhang *et al.*^33^, for Bi_2_Se_3_ thicknesses beneath 6 nm the surface state at the two interfaces can interfere, disrupting the bandstructure. In this instance, direct communication between top and bottom surface should be possible, without passing through the bulk. If the TSS at each surface in these samples have retreated by ~ 1 nm due to FM proximity or surface roughness^34^, then this could explain the coupling observed for 4 nm and 8 nm, while the distinct states at 20 nm do not allow this. The VNA-FMR experiments then probe the spin sink properties of the bulk, which has great capacity to absorb angular momentum, arising from its large spin-orbit coupling.

In summary, these experimental results demonstrate that TIs hold great promise for the field of spintronics. They display an unusual thickness dependence to their Gilbert damping, demonstrating a high capacity to absorb angular momentum, and – if the process is reversed – to generate a significant spin transfer torque. TR-XMCD experiments reveal that transfer of angular momentum between ferromagnetic layers in a spin valve structure is possible, either through conventional spin pumping or possibly through a direct coupling of the topological surface state. As these effects can be observed at room temperature and low magnetic fields, TIs are particularly well suited to future device applications, as well as being a fertile ground for investigation of fundamental physical phenomena.

## Methods

### Ferromagnetic Resonance Measurements

VNA-FMR measurements were performed using a Rhode and Schwartz ZVB20 vector network analyser. Samples were mounted face-down on a coplanar waveguide of characteristic impedance 50 Ω and placed in an octupole electromagnet, capable of applying a field of up to 0.5 T in any direction. Real and imaginary components of the microwave transmission parameter, *S*_12_, were measured as a function of field vector (strength and angle) and frequency. The resulting resonances were fitted using asymmetric Lorentzians to extract resonance frequency and linewidth. All measurements were performed at room temperature.

### Time Resolved X-ray Magnetic Circular Dichroism

TR-XMCD measurements were performed on beamline I10 at Diamond Light Source (UK) and beamline 4.0.2 at the Advanced Light Source (US). TR-XMCD offers an element (and thus layer-) specific time-resolved measurement of magnetisation alignment, allowing mapping of the precessional dynamics of each FM layer on the picosecond timescale. The sample is mounted on a CPW and driven by an applied RF field while under a DC bias field. The XMCD effect is then used as an element specific probe of magnetisation, as the size of the effect scales as the cosine of the angle between the incident x-ray helicity vector and the magnetisation alignment. By synchronising the driving RF with a multiple of the master oscillator clock of the synchrotron, the magnitude of the XMCD can be probed as a function of delay between RF excitation (pump) and x-ray bunch arrival (probe). The x-ray absorption in transmission was detected using luminescence of the MgO substrate. All TR-XMCD measurements were performed at the Ni and Co *L*_3_ edges.

## Author Contributions

A.A.B., G.v.d.L. and T.H. conceived the idea and A.A.B., L.C.M. and T.H. fabricated the device structure. G.v.d.L. and A.I.F. developed the TR-XMCD instrumentation. A.A.B. and A.I.F. performed the VNA-FMR measurements, all authors took part in the TR-XMCD experiments, A.A.B. and A.I.F. analysed the data. A.A.B. and T.H. wrote the paper with comments and input from all authors. All authors contributed to the discussions.

## Supplementary Material

Supplementary Information Supplementary material

## Figures and Tables

**Figure 1:**
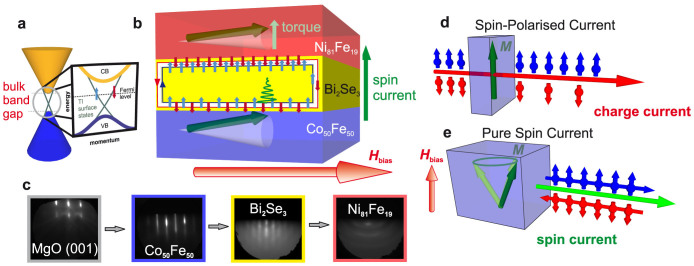
Schematic of the FM-TI-FM heterostructure and key concepts of spin pumping. (a), Bandstructure of a TI, showing the valence (blue) and conduction (orange) bands, with the spin-locked surface state crossing the bulk bandgap. (b), Schematic of the device structure, showing the TI Bi_2_Se_3_ placed between two FM layers. The surface state is indicated by up- and down-arrows, representing counter-propagating spin-momentum locked conduction. The precession of magnetisation excited around the static bias field drives a pure spin current from the Co_50_Fe_50_ through the Bi_2_Se_3_ into the Ni_81_Fe_19_, exerting a spin transfer torque. (c), RHEED images of the growth of each layer. (d),(e), Illustration of the difference between a spin polarised current and a pure spin current. Note the similarity between counter-propagation of spins in a pure spin current and the counter propagating conduction channels in the TSS.

**Figure 2:**
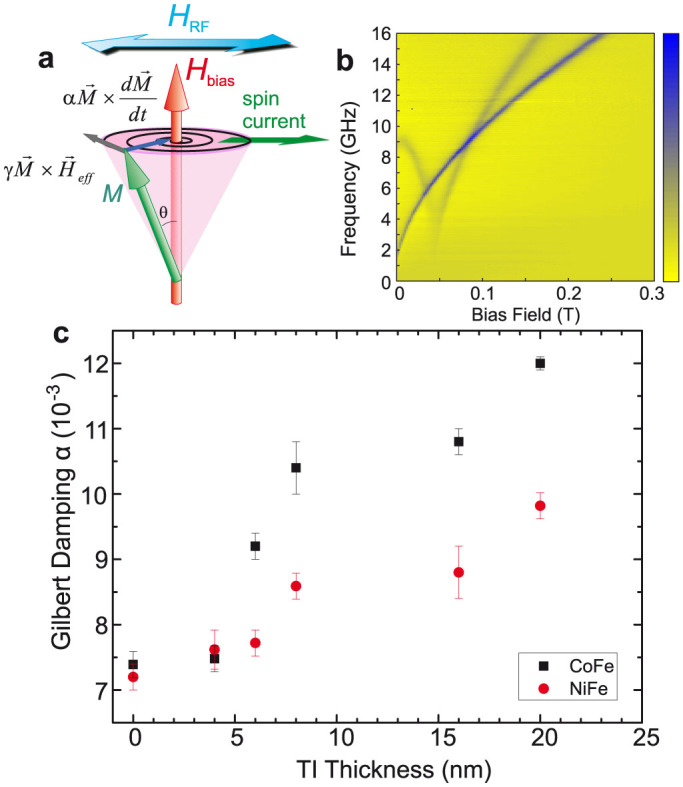
Gilbert damping increases as a function of TI interlayer thickness. (a), Diagram of the forces acting on the magnetisation of a material undergoing FMR, showing excitation field (***H***_RF_), static bias field (***H***_B_) and precessing magnetisation vector (***M***). ***M*** × ***H****_eff_* acts as the driving force of precession, while 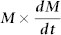 “brakes” the magnetisation back towards the equilibrium condition according to the total damping, *α*. (b), Field-frequency-transmission map showing on-resonance absorption of the heterostructure. Blue indicates high absorption, when the resonance condition is met. The bias field is applied parallel to the [100] axis of Co_50_Fe_50_, an in-plane hard axis, so the lower branch of the Kittel curve corresponds to magnetisation canted along the in-plane easy axis, the upper branch to the magnetisation collinear with the bias field. (c), Calculated damping factor as a function of thickness of the TI interlayer for Co_50_Fe_50_ (black) and Ni_81_Fe_19_ (red) layers. Error bars represent the uncertainty on linear fits to linewidth as a function of frequency.

**Figure 3:**
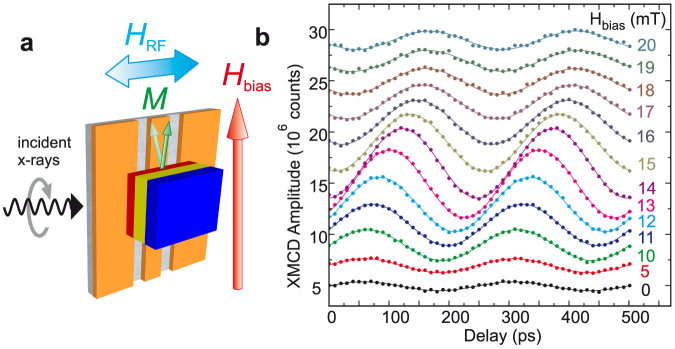
Time-resolved precession of magnetisation of Ni. (a), Illustration of the experimental configuration for the measurement of TR-XMCD. The sample is placed face-down on a CPW and incident circularly polarised x-rays pass through a hole in the signal line. The magnetisation of the stack about *H*_bias_ is driven by the RF field, *H*_RF_. The cone angle of precession is exaggerated for clarity; the typical magnitude of precession is ~ 1°. (b), TR-XMCD data for Ni_81_Fe_19_ continuously driven at 4 GHz for the Co_50_Fe_50_(30)/Bi_2_Se_3_(8)/Ni_81_Fe_19_(30) sample (thicknesses in nm). Varying static bias field indicated by colour of lines, offset for clarity, showing increase in amplitude and phase shift across resonance at 14 mT. Solid lines are sine curve fits to the data.

**Figure 4:**
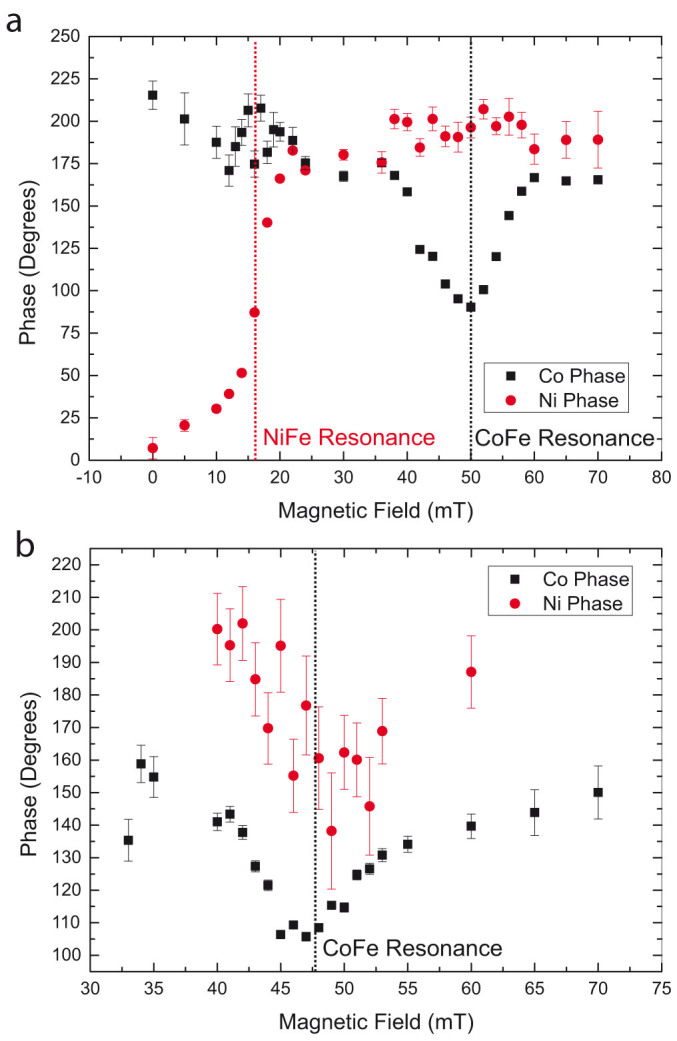
Phase variation across resonance. Phase of precession of magnetisation for Ni_81_Fe_19_ (red circles) and Co_50_Fe_50_ (black squares) layers at 4 GHz driving frequency for *t*_TI_ = 4 nm and 8 nm ((a),(b), respectively). Dashed lines show the positions of the resonance amplitude peak The drop and recovery in phase across the Co mode is caused by the superposition of the two modes, corresponding to canted and collinear magnetisation, as can be seen in Fig. 2b. Error bars arise from uncertainty on fits to the time-resolved precession, see Fig. 3b.
