# Supplementary material for: Spin pumping in Ferromagnet-Topological Insulator-Ferromagnet Heterostructures
Source: Sci Rep. 2015 Jan 20;5:7907. doi: 10.1038/srep07907 (PMC4298741; doi:10.1038/srep07907)
Supplement: Supplementary Information — Supplementary material [file srep07907-s1.pdf]

Spin pumping in Ferromagnet-Topological Insulator-Ferromagnet Heterostructures Supplementary Information

A.A. Baker,[1,2] A.I. Figueroa,[2] L.J. Collins-McIntyre,[1] G. van der Laan,[2] and T. Hesjedal[1,a)]

[1)] *Department of Physics, Clarendon Laboratory, University of Oxford, Oxford, OX1 3PU, UK*

[2)] *Magnetic Spectroscopy Group, Diamond Light Source, Didcot, OX11 0DE, UK*

[a)] Corresponding author. Email: thorsten.hesjedal@physics.ox.ac.uk

I. THEORY OF SPIN PUMPING

Spin dynamics of an ultrathin film are well described by the Landau-Lifshitz-Gilbert equation of motion:

$$\frac{\partial \boldsymbol{M}}{\partial t} = -\gamma \left[\boldsymbol{M} \times \boldsymbol{H}_{\text{eff}}\right] + \alpha \left[\boldsymbol{M} \times \frac{\partial \boldsymbol{u}}{\partial t}\right], \tag{1}$$

where $\boldsymbol{u}$ is the unit vector of the direction of magnetisation, $\boldsymbol{M}$; γ the gyromagnetic ratio and α the dimensionless Gilbert damping constant, which parametrises energy loss through such mechanisms as coupling to the lattice or two-magnon scattering.[1] As the magnetisation of a ferromagnetic layer precesses on resonance it acts as a spin battery, generating a pure spin current transverse to the axis about which it precesses. Therefore, when the ferromagnetic (FM) layer is thicker than the ferromagnetic coherence length, a pure spin current can be driven into an adjacent non-magnetic (NM) layer. The pumped spin momentum that enters the NM layer, $\boldsymbol{I_{sp}}$, is determined by the spin mixing conductance, $\mathrm{g}_{\uparrow\downarrow}$ as:[2]

$$\boldsymbol{I_{sp}} = \frac{\hbar}{4\pi}\text{Re}(g_{\uparrow\downarrow})\left[\boldsymbol{u} \times \frac{\partial \boldsymbol{u}}{\partial t}\right]. \tag{2}$$

If the spin-flip relaxation rate in the adjacent NM layer is smaller than the pumping rate a total spin angular momentum $\boldsymbol{s}$ builds up in the NM layer, and spin-backflow occurs. This leads to a backflow current, back into the FM layer:

$$I_{back} = \frac{g_{\uparrow\downarrow}}{2\pi N}\left[\boldsymbol{s} - \boldsymbol{m}(\boldsymbol{m}) \cdot \boldsymbol{s}\right], \tag{3}$$

with N the one-spin density of states. Therefore, the total spin momentum leaving the FM layer is reduced by the amount flowing back into it from the normal metal. The penetration of the spin current into the NM is then:

$$\delta_{sp} = D \cdot \tau_{sf} = v_{\text{F}}\sqrt{\tau_{sf}\tau_m/3}, \tag{4}$$

where D is the diffusion coefficient in the normal metal, v_{F} the Fermi velocity in the NM layer, and τ_{sf} and τ_m the NM layer's spin-flip and momentum scattering times, respectively.

The increased flow of spin momentum out of the FM layer then acts as another channel for energy loss, leading to an observable increase in damping. Since this damping is linear with resonant frequency it can be described in the same terms as Gilbert damping, and its contribution isolated by comparison with bare FM layers. In a FM/NM system the

spin-pumping contribution to damping is:

$$\alpha_{sp} = \left[1 - \frac{(1+e^{-2kd})\frac{1}{2}v_{\mathrm{F}}}{\left(Dk + \frac{1}{2}v_{\mathrm{F}} + e^{-2kd}\right)\left(\frac{1}{2}v_{\mathrm{F}} - Dk\right)}\right] \times \frac{g\mu_{\mathrm{B}}}{4\pi M_s}\tilde{g}_{\uparrow\downarrow}\frac{1}{d}, \tag{5}$$

where $k = 1/\delta_{sp}$ and d the thickness of the FM layer. As the thickness of the NM layer increases, the spin current that it can absorb also increases. This manifests as an increase in damping. However, once $t_{NM} > \delta_{sp}$, α_{sp} saturates to its maximum value.

In the case of a FM1/NM/FM2 structure, the second FM layer acts as a spin sink for the spin current driven out of the first FM layer. The absorbed spin current exerts a torque on the static magnetisation in FM2, which can lead to precession of magnetisation even when the resonance condition is not met. The addition of a second scattering interface and a high-efficiency spin sink modifies the spin pumping equations significantly. In this case the LLG becomes:[3]

$$\frac{\partial \boldsymbol{m}_i}{\partial t} = -\gamma\left[\boldsymbol{m}_i \times \boldsymbol{H}^i_{\mathrm{eff}}\right] + \alpha^0_i\left[\boldsymbol{m}_i \times \frac{\partial \boldsymbol{m}_i}{\partial t}\right] + \alpha^{sp}_i\left[\boldsymbol{m}_i \times \frac{\partial \boldsymbol{m}_i}{\partial t} - \boldsymbol{m}_j \times \frac{\partial \boldsymbol{m}_j}{\partial t}\right], \tag{6}$$

where the subscript denotes the magnetic layer number, α^0 the intrinsic Gilbert damping parameter and α^{sp} an additional damping due to spin pumping. The average magnetisation is then damped as before, by α^0, while the difference experiences an increased damping $\alpha = \alpha^0 + \alpha^{\mathrm{sp}}_1 + \alpha^{\mathrm{sp}}_2$. The additional damping terms are defined as

$$\alpha_{sp} = \left[1 - \frac{\left[\left(Dk + \frac{1}{2}v_{\mathrm{F}}\right) + e^{-2kd}\left(Dk - \frac{1}{2}v_{\mathrm{F}}\right)\right]\frac{1}{2}v_{\mathrm{F}}}{\left(Dk + \frac{1}{2}v_{\mathrm{F}}\right)^2 + e^{-2kd}\left(Dk - \frac{1}{2}v_{\mathrm{F}}\right)^2}\right] \times \frac{g\mu_{\mathrm{B}}}{4\pi M_s}\tilde{g}_{\uparrow\downarrow}\frac{1}{d}, \tag{7}$$

which, in the limit of ballistic transport, goes to:[4]

$$\alpha_{sp} = \frac{g\mu_{\mathrm{B}}}{4\pi M}\tilde{g}_{\uparrow\downarrow}\frac{1}{d}. \tag{8}$$

Damping is highest when the spin current can cross the spacer layer and be efficiently absorbed by the second FM layer. As the thickness of the spacer layer increases the spin pumping decreases due to increasing backflow. The increase in damping reaches its minimum

value when the spacer layer is thicker than the spin coherence length, and no current reaches FM2.[5]

While this theory adequately describes pumping into the bulk states of a TI, which are essentially a normal insulator with high spin-orbit coupling, it does not cover pumping into the surface state of the TI. Here an additional damping term arises due to the exchange interaction with the spin-polarised surface states of the TI, which acts as a braking torque on the precessing magnetisation in the ferromagnet. Yokoyama *et al.*[6] proposed an exchange field:

$$H_{\rm ex} = -E_{\rm ex} \int d\boldsymbol{r}\boldsymbol{n}(\boldsymbol{r}) \cdot \hat{\boldsymbol{\sigma}}(\boldsymbol{r}), \tag{9}$$

where $E_{\rm ex}$ is the exchange coupling energy, $\hat{\boldsymbol{\sigma}}(\boldsymbol{r})$ (twice) the electron spin density and $\boldsymbol{n}(\boldsymbol{r})$ a unit vector pointing in the direction of the localized spins $\boldsymbol{S} = S\boldsymbol{n}$. The torque induced by this interaction is:[7]

$$T_{surface} = \gamma \vec{\boldsymbol{M}} \times \frac{\mu_0 E_{ex} n_{\rm FM} S}{M} \langle \vec{\boldsymbol{\sigma}} \rangle, \tag{10}$$

with $n_{\rm FM}$ the number of atoms per unit volume in the FM layer. The electron spin density can also be written:

$$\langle \vec{\boldsymbol{\sigma}} \rangle = \frac{\mu_{\rm H} E_{\rm ex}}{e v_{\rm F^2}} \frac{S}{M} \frac{d\vec{\boldsymbol{M}}}{dt}, \tag{11}$$

where μ_H is the surface electron mobility and v_F is the surface electron Fermi velocity. This term, with its time derivative of magnetisation, is reminiscent of the LLG in equation 1. Substituting back into the torque term we see the equation is nothing other than a damping term:

$$T_{surface} = \frac{\mu_{\rm H} E_{\rm ex}^2}{\hbar e v_{\rm F^2}} \frac{S}{M} \vec{\boldsymbol{M}} \times \frac{d\vec{\boldsymbol{M}}}{dt} \tag{12}$$

with damping factor:

$$\alpha_{surface} = \frac{\mu_{\rm H} E_{\rm ex}^2}{\hbar e v_{\rm F^2}} \frac{S}{M}. \tag{13}$$

Therefore there exist three components of the damping factor for a FM adjacent to a TI:

$$\alpha_{\rm total} = \alpha_{\rm bare} + \alpha_{\rm bulk} + \alpha_{\rm surface} \tag{14}$$

where $\alpha_{\rm bare}$ is damping of an isolated ferromagnetic layer, $\alpha_{\rm bulk}$ damping due to spins pumped into the bulk of the TI, and $\alpha_{\rm surface}$ damping arising due to coupling to the surface state. One can therefore expect a significant enhancement of damping in such samples.

Finally, we note also the exchange coupling term of the Hamiltonian of the surface state:[8]

$$\hat{H_{\rm ex}} = v_{\rm F} \cdot \vec{\sigma}(\vec{\boldsymbol{a}} \times \hat{z}) - \frac{E_{\rm ex}}{M} \vec{\boldsymbol{\sigma}} \cdot \boldsymbol{n}(\boldsymbol{r}), \tag{15}$$

with $\hat{z}$ out of the plane of the film. This incorporates the effective vector potential:[7]

$$\vec{\boldsymbol{a}} = \frac{E_{ex}}{ev_{\mathrm{F}}} \frac{S}{M} \hat{z} \times \vec{\boldsymbol{M}}. \tag{16}$$

This will affect the free energy of the ferromagnet, and therefore cause a shift in resonance frequency with respect to a bare ferromagnet.

II. SAMPLE FABRICATION

Samples were prepared using molecular beam epitaxy (MBE) under ultra-high vacuum (UHV) conditions of better than 5×10^{-10} Torr. Sources were calibrated using a quartz crystal microbalance and beam-flux monitor. First, the MgO substrate was annealed at 500°C to clean the surface and improve the crystalline quality. Next, 30 nm of $Co_{50}Fe_{50}$ was co-evaporated from Fe and Co electron-beam evaporators. The substrate temperature was held at 300°C during this stage. Epitaxial growth of $Co_{50}Fe_{50}$ is possible despite the lattice mismatch with MgO due to a rotation of crystal domains. We observed strong streaks and Kikuchi lines in the RHEED patterns, indicative of high crystalline quality. Samples were then transferred into a chalcogenide MBE for growth of the Bi_2Se_3 layers. The $Co_{50}Fe_{50}$ surface was again annealed at 300°C to ensure the surface was high quality; this was checked by RHEED before growth. The Bi and Se were then evaporated from Knudsen cells for stoichiometric growth of Bi_2Se_3. Substrate temperature was 200°C. RHEED patterns reveal the formation of a crystalline mosaic domain pattern. Bi_2Se_3 thickness was varied between 4 - 20 nm. The samples was then transferred back to the metals MBE chamber, and a top FM layer of $Ni_{81}Fe_{19}$ (30 nm) deposited at room temperature ($\sim$ 300 K), to avoid damaging the Bi_2Se_3 layer structure. This leads to the formation of polycrystalline $Ni_{81}Fe_{19}$, which is desirable. Samples were then capped with either 10 nm Cr or 5 nm Cu, to prevent oxidation.

REFERENCES

[1]Woltersdorf, G., Buess, M., Heinrich, B. & Back, C. Time resolved magnetiszation dynamics of ultrathin Fe (001) films: spin-pumping and two-magnon scattering. *Phys. Rev. Lett.* **95**, 037401 (2005).

[2]Tserkovnyak, Y., Brataas, A., Bauer, G. E. & Halperin, B. I. Nonlocal magnetization dynamics in ferromagnetic heterostructures. *Rev. Mod. Phys.* **77**, 1375 (2005).

[3]Heinrich, B. *et al.* Dynamic exchange coupling in magnetic bilayers. *Phys. Rev. Lett.* **90**, 187601 (2003).

[4]Marcham, M. *et al.* Phase-resolved x-ray ferromagnetic resonance measurements of spin pumping in spin valve structures. *Phys. Rev. B* **87**, 180403 (2013).

[5]Kardasz, B. & Heinrich, B. Ferromagnetic resonance studies of accumulation and diffusion of spin momentum density in Fe/Ag/Fe/GaAs (001) and Ag/Fe/GaAs (001) structures. *Phys. Rev. B* **81**, 094409 (2010).

[6]Yokoyama, T., Zang, J. & Nagaosa, N. Theoretical study of the dynamics of magnetization on the topological surface. *Phys. Rev. B* **81**, 241410 (2010).

[7]Shiomi, Y. *et al.* Bulk topological insulators as inborn spintronics detectors. *arXiv preprint: arXiv:1312.7091* (2013).

[8]Garate, I. & Franz, M. Inverse spin-galvanic effect in the interface between a topological insulator and a ferromagnet. *Phys. Rev. Lett.* **104**, 146802 (2010).
